# Association of Immune‐Related Adverse Events and the Efficacy of Immune Checkpoint Inhibitors in Non‐Small Cell Lung Cancer: Adjusting for Immortal Time Bias

**DOI:** 10.1111/1759-7714.70261

**Published:** 2026-03-16

**Authors:** Yoshiki Kuwabara, Hiroyuki Ohya, Maiko Osawa, Kota Shiraishi, Itsuka Matsumoto, Shigeru Ishii, Shin Yokosuka, Masatoshi Abe, Tomoyuki Takahashi, Yuichiro Kawano, Hiroaki Nishimura, Maiko Toda‐Sasaki, Yumiko Kobayashi‐Ogawa, Satoshi Kikuchi, Yusuke Hirata, Kosuke Sakai, Hiroyuki Kyoyama, Gaku Moriyama, Yohei Kawasaki, Nobuyuki Koyama, Kazutsugu Uematsu

**Affiliations:** ^1^ Department of Pulmonary Medicine, Saitama Medical Center Saitama Medical University Kawagoe Japan; ^2^ Department of Pharmacy, Saitama Medical Center Saitama Medical University Kawagoe Japan; ^3^ Department of Biostatistics, Graduate School of Medicine Saitama Medical University Saitama Japan

**Keywords:** immortal time bias, immune checkpoint inhibitors, immune‐related adverse events, nonsmall cell lung cancer

## Abstract

**Background:**

Associations between immune‐related adverse events (irAEs) and survival outcomes in non‐small cell lung cancer (NSCLC) patients treated with immune checkpoint inhibitors (ICIs) remain controversial, partly due to inconsistencies in dealing with immortal time bias (ITB). To address this, we adjusted for ITB in this single‐center retrospective study.

**Methods:**

We included 129 patients with advanced NSCLC receiving ICIs as first‐line therapy and assessed associations between irAE development and progression‐free survival (PFS) and overall survival (OS). To mitigate ITB, we performed landmark analyses at 30, 42, and 75 days and used multivariable Cox models with irAEs as a time‐dependent covariate.

**Results:**

During a median follow‐up of 9.7 months, 58 (45.0%) patients developed irAEs. Unadjusted analyses showed a significant association between irAEs and longer PFS (hazard ratio [HR] 0.55; *p* < 0.01) and OS (HR 0.42; *p* < 0.01), but this was no longer evident after adjusting for ITB. Specifically, landmark analyses revealed no significant survival differences, except for PFS at the 42‐day landmark. The time‐dependent Cox model confirmed no significant association between irAE occurrence and PFS (HR 1.16) or OS (HR 0.93). In contrast, good Eastern Cooperative Oncology Group performance status at baseline was an independent predictor of improved survival.

**Conclusion:**

After appropriate adjustment for ITB, irAE development was not associated with improved survival in NSCLC patients treated with ICIs. Baseline performance status, rather than irAE occurrence, remains a more critical prognostic factor for these patients. The relationship between irAE development and clinical outcome should be evaluated with consideration of ITB.

## Graphical abstract

Immortal time bias (ITB)–adjusted landmark and time‐dependent Cox analyses showed that immune‐related adverse events (irAEs) were not associated with improved survival in NSCLC patients receiving first‐line immune checkpoint inhibitors. ECOG performance status remained the key prognostic factor. The irAE–outcome relationship should be evaluated with consideration of ITB.

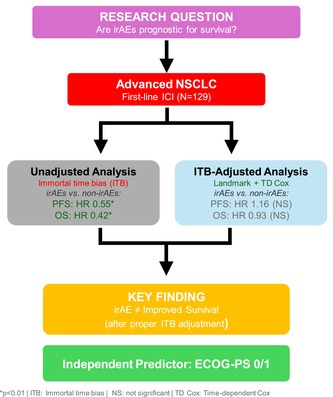

## 1 Introduction

Immune checkpoint inhibitors (ICIs) that target programmed cell death protein 1 (PD‐1), programmed death‐ligand 1 (PD‐L1), or cytotoxic T‐lymphocyte‐associated protein 4 (CTLA‐4) enhance the antitumor immune response [1] and bring about significant clinical benefits in the treatment of several cancers. ICIs are also approved for patients with advanced nonsmall cell lung cancer (NSCLC) in various settings [2].

Despite their clinical benefits, ICIs induce a unique spectrum of side effects, known as immune‐related adverse events (irAEs). Although the exact pathophysiology remains unclear, potential mechanisms underlying the development of irAEs include T cell activity against shared antigens, the effects of autoantibodies, increased levels of inflammatory cytokines, and direct complement activation by anti‐CTLA‐4 antibodies [3]. Although the occurrence of irAEs may indicate host immune system activation, because of conflicting published data it remains contentious as to whether this translates to enhanced antitumor immunity and improved clinical outcomes in NSCLC. Given this context, it has been hypothesized that the occurrence of irAEs reflects immune system reinvigoration, potentially leading to greater clinical benefit from ICIs [4]. Several studies have reported a positive association between irAEs and survival in NSCLC patients treated with ICIs, whereas others found no significant difference in progression‐free survival (PFS) or overall survival (OS) between groups with and without irAEs. Some meta‐analyses suggest a positive correlation between irAEs and improved PFS or OS [5, 6]. However, these meta‐analyses may include studies susceptible to immortal time bias (ITB). Because irAEs typically develop after initiation of ICIs, patients must survive long enough to experience them, avoiding early progression or death. Analyzing irAE occurrence as a fixed baseline characteristic without accounting for this “immortal” time can lead to biased estimates favoring the irAE group [7, 8, 9]. Thus, previous studies [10, 11, 12] and meta‐analyses [5, 6] that did not explicitly address ITB should be interpreted with caution.

To accurately evaluate associations between the development of irAEs and clinical outcomes while accounting for ITB, landmark analyses or time‐dependent Cox models have been used. Landmark analyses offer visual interpretability via Kaplan–Meier curves; however, they require careful selection of time points and may have limited statistical power. Time‐dependent Cox models are generally considered more statistically powerful because they use all follow‐up data from cohort entry [7, 8]. Studies that have addressed ITB using the landmark approach have reported conflicting results regarding the prognostic impact of the occurrence of irAEs. For example, some studies have reported a positive correlation between the occurrence of irAEs and PFS or OS [13, 14, 15, 16, 17], whereas others have reported no association [18, 19, 20, 21]. Many studies using time‐varying Cox proportional hazards models have reported a positive correlation between irAE occurrence and PFS or OS [17, 18, 22]. This discrepancy is often explained by the statistical power of the study. Therefore, definitive evidence regarding the prognostic impact of irAEs, specifically accounting for ITB, is still needed.

In the present study we evaluated the association between irAEs and long‐term clinical outcomes in patients with advanced NSCLC treated with ICIs as first‐line therapy, which is routine clinical practice at Saitama Medical Center, Saitama Medical University. We used landmark analyses and a time‐dependent Cox proportional hazards model to address ITB.

## 2 Methods

### 2.1 Patients

All patients with advanced NSCLC who had undergone first‐line therapy including anti‐PD‐1, anti‐PD‐L1, or anti‐CTLA‐4 antibodies at Saitama Medical Center, Saitama Medical University between January 2021 and December 2024 were retrospectively enrolled in the study. Patients with other types of cancer, or those who had previously received molecular targeted therapy were excluded.

### 2.2 Procedures

Clinical information was retrospectively collected from electronic medical records at Saitama Medical Center. Data collection was censored as of December 31, 2024. Treatment response was classified according to the Response Evaluation Criteria in Solid Tumors (RECIST) v1.1. The objective response rate (ORR) was defined as the proportion of patients achieving a partial or complete response. PFS was defined as the time from treatment initiation to disease progression or death. OS was defined as the time from treatment initiation to death from any cause or last follow‐up. IrAEs were defined as any adverse events considered by the attending physician to be possibly or definitely related to ICI. The diagnosis was confirmed after excluding other potential etiologies, such as infection, and often required consultation with relevant specialists. The severity of irAEs was graded according to the Common Terminology Criteria for Adverse Events (CTCAE) [23].

### 2.3 Statistical Analyses

The significance of differences in characteristics between the irAE and non‐irAE groups, including sex, Eastern Cooperative Oncology Group performance status (ECOG‐PS), smoking history, PD‐L1 status, driver mutation status, the presence of brain metastasis, treatment response, type of ICI treatment, and combination with chemotherapy was evaluated using the chi‐squared test or Fisher's exact test. Chi‐squared tests were used for comparisons of categorical variables; however, Fisher's exact test was used when the expected cell count was less than 5. The significance of differences in age between the irAE and non‐irAE groups was assessed using the Mann–Whitney U test.

ORR was evaluated by logistic regression analysis, adjusting for covariates including sex, age, driver mutation status, and ECOG‐PS. PFS and OS were estimated by the Kaplan–Meier method, and differences in PFS and OS between groups were assessed using log‐rank tests. For comparisons among three groups, pairwise log‐rank tests were performed with a Holm correction if the overall log‐rank test was significant. To mitigate ITB, landmark analyses for PFS and OS were conducted at 30, 42, and 75 days after treatment initiation. These specific time points were selected to reflect key moments in treatment and irAE development. Specifically, 30 days corresponds approximately to the 25th percentile of time to first irAE onset, 75 days represents the median time to first irAE onset in the present study cohort, and 42 days reflects the standard interval for initial evaluation of treatment response. These time points included only patients who were progression‐free or alive at the respective landmark time.

In addition, Cox hazard ratios (HRs) and 95% confidence intervals (CIs) were estimated by the Cox proportional hazards regression model. Factors associated with PFS (including sex, age, driver mutation status, ECOG‐PS, PD‐L1 status, and with or without chemotherapy) or OS (including sex, age, driver mutation status, and ECOG‐PS) were determined using the multivariable Cox proportional hazards model. To further account for ITB, Cox models incorporated irAE occurrence as a time‐dependent covariate. This covariate was coded as “0” before the onset of the first irAE and “1” thereafter for patients experiencing an irAE; it remained “0” throughout follow‐up for those without irAEs [9].

To assess the statistical power of the study, a post hoc power calculation was performed. Since calculating the power for a time‐dependent Cox proportional hazards model is complex, we adopted an alternative approach using Schoenfeld's formula. The calculation estimated the probability of detecting a specific hazard ratio (HR) based on the total number of events (OS: *E* = 46, PFS: *E* = 79) and the proportion of the irAE group (*p* = 58/129 ≈ 0.45). We defined a clinically relevant effect size by assuming hazard ratios of HR = 0.70 and HR = 0.60 for the primary analysis, with a two‐sided significance level of *α* = 0.05.

### 2.4 Ethical Considerations

The study protocol was approved by the Ethics Board of Saitama Medical Center, Saitama Medical University (Approval no. 2024‐219). This study is a retrospective study, and patient consent was obtained through the facility's opt‐out procedure.

## 3 Results

### 3.1 Patient Characteristics

**FIGURE 1:**
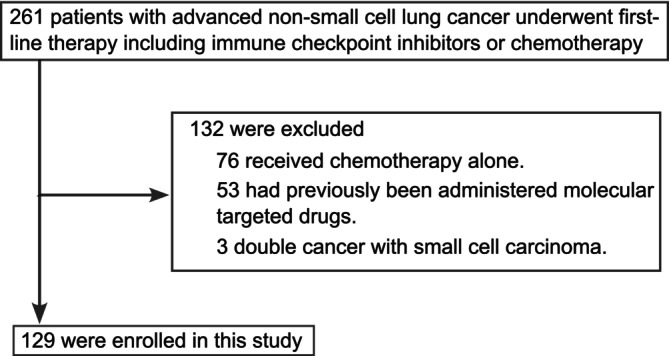
CONSORT diagram for the selection of patients with nonsmall cell lung cancer treated with first‐line therapy.

During the study period, 261 patients with advanced NSCLC underwent first‐line therapy at Saitama Medical Center, Saitama Medical University. Of those, 132 patients were excluded from this analysis because they did not meet the enrollment criteria: 76 patients received chemotherapy alone; 53 patients had previously received molecular targeted therapy; and three patients suffered from double cancer with small cell carcinoma. Consequently, 129 patients who received anti‐PD‐1, anti‐PD‐L1, or anti‐CTLA4 were included in this single institution's retrospective study (Figure 1). Of those, 58 developed at least one irAE during the observation period. Based on the occurrence of at least one irAE, patients were divided into an irAE group (*n* = 58; 45.0%) and a non‐irAE group (*n* = 71; 55.0%). Baseline characteristics were generally balanced between these two groups (Table 1).

**TABLE 1:** Patient characteristics.

	All patients (*n* = 129)	No irAEs (*n* = 71)	irAEs (*n* = 58)	*p*
Age (years)	70 (34–85)	70 (42–85)	71 (34–85)	0.62
Sex				1
Male	106 (82.2)	58 (81.7)	48 (82.8)	
Female	23 (17.8)	13 (18.3)	10 (17.2)	
ECOG‐PS				0.224
0, 1	110 (85.3)	58 (81.7)	52 (89.7)	
≥ 2	19 (14.7)	13 (18.3)	6 (10.3)	
Smoking status				0.381
Never	13 (10.1)	9 (12.7)	4 (6.9)	
Current or former	116 (89.9)	62 (87.3)	54 (93.1)	
Histology				0.55
Squamous	24 (18.6)	15 (21.1)	9 (15.5)	
Adenocarcinoma	96 (74.4)	50 (70.4)	46 (79.3)	
Other	9 (7.0)	6 (8.5)	3 (5.2)	
PD‐L1 status				0.371
≥ 50%	56 (46.3)	28 (41.8)	28 (51.9)	
1%–49%	23 (19.0)	12 (17.9)	11 (20.4)	
< 1%	42 (34.7)	27 (40.3)	15 (27.8)	
Driver mutation status				1
No	96 (74.4)	53 (74.6)	43 (74.1)	
Yes	33 (25.6)	18 (25.4)	15 (25.9)	
Brain metastasis				0.186
No	103 (79.8)	60 (84.5)	43 (74.1)	
Yes	26 (20.2)	11 (15.5)	15 (25.9)	
RECIST				0.447
CR	6 (5.2)	3 (5.0)	3 (5.5)	
PR	42 (36.5)	18 (30.0)	24 (43.6)	
SD	43 (37.4)	24 (40.0)	19 (34.5)	
PD	24 (20.9)	15 (25.0)	9 (16.4)	
Type of ICI treatment				0.295
PD‐1 inhibitors	95 (73.6)	52 (73.2)	43 (74.1)	
PD‐L1 inhibitors	27 (20.9)	17 (23.9)	10 (17.2)	
CTLA‐4 inhibitors	7 (5.4)	2 (2.8)	5 (8.6)	
With or without chemotherapy				0.549
ICI only	34 (26.4)	17 (23.9)	17 (29.3)	
With chemotherapy	95 (73.6)	54 (76.1)	41 (70.7)	
irAE grade				
Grade 1/2			33 (56.9)	
Grade 3–5			25 (43.1)	
No. organs involved				
1			44 (75.9)	
≥ 2			14 (24.1)	
Use of corticosteroids				
Yes			31 (53.5)	
No			27 (46.5)	

*Note:* Unless indicated otherwise, data are given as the median (minimum–maximum) or *n* (%).

Abbreviations: CR, complete response; CTLA‐4, cytotoxic T‐lymphocyte‐associated protein 4; ECOG‐PS, Eastern Cooperative Oncology Group performance status; ICI, immune checkpoint inhibitor; irAEs, immune‐related adverse events; PD, progressive disease; PD‐1, programmed cell death protein 1; PD‐L1, programmed death‐ligand 1; PR, partial response; RECIST, Response Evaluation Criteria in Solid Tumors; SD, stable disease.

### 3.2 Incidence and Spectrum of irAEs

**FIGURE 2:**
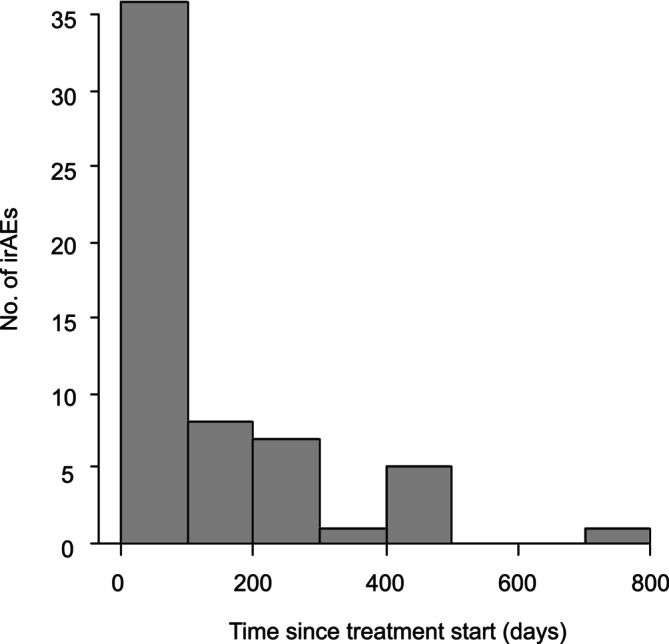
Distribution of the onset of immune‐related adverse events (irAEs) after the start of treatment.

In the irAE group, 58 patients experienced a total of 77 distinct irAEs, indicating multiple‐organ involvement in some patients. The median time to onset of the first irAE was 77 days (minimum–maximum 0–749 days; Figure 2). Among the 77 irAE events, pneumonitis (*n* = 25; 32.5%) was the most frequent, followed by hepatic toxicity (*n* = 15; 19.5%), thyroid dysfunction (*n* = 6; 7.8%), diarrhea/colitis (*n* = 5; 6.5%), skin toxicity (*n* = 5; 6.5%), and other disorders. Patients with irAEs were divided into two groups according to severity: Grade 1/2 (*n* = 44; 57.1%) and Grade 3–5 (*n* = 33; 42.9%). Among the Grade 1/2 events, pneumonitis (*n* = 18; 23.4%) was again the most frequent, followed by hepatic toxicity (*n* = 6; 7.8%), and thyroid dysfunction (*n* = 5; 6.5%). Among the Grade 3–5 events, hepatic toxicity (*n* = 9; 11.7%) was the most frequent, followed by pneumonitis (*n* = 7; 9.1%), and diarrhea/colitis (*n* = 5; 6.5%; Table 2).

**TABLE 2:** Immune‐related adverse events according to severity.

irAEs	Total	irAE severity	
		Grade 1/2	Grade 3–5
All irAEs	77 (100)	44 (57.1)	33 (42.9)
Pneumonitis	25 (32.5)	18 (23.4)	7 (9.1)
Hepatic toxicity^a^	15 (19.5)	6 (7.8)	9 (11.7)
Thyroid dysfunction^b^	6 (7.8)	5 (6.5)	1 (1.3)
Diarrhea/colitis	5 (6.5)	0 (0)	5 (6.5)
Skin toxicity^c^	5 (6.5)	4 (5.2)	1 (1.3)
Adrenal/pituitary dysfunction	4 (5.2)	1 (1.3)	3 (3.9)
Neuromuscular toxicity	5 (6.5)	3 (3.9)	2 (2.6)
Renal toxicity	3 (3.9)	1 (1.3)	2 (2.6)
Pancreatic toxicity^d^	2 (2.6)	2 (2.6)	0 (0)
Infusion reaction	2 (2.6)	2 (2.6)	0 (0)
Arthritis	1 (1.3)	1 (1.3)	0 (0)
Myocarditis	1 (1.3)	0 (0)	1 (1.3)
Others	3 (3.9)	1 (1.3)	2 (2.6)

*Note:* Data are presented as *n* (%).

^a^ Hepatic toxicity includes increased alanine aminotransferase or aspartate aminotransferase, increased bilirubin levels, and hepatitis.

^b^ Thyroid dysfunction includes hypo‐ and hyperthyroidism.

^c^ Skin toxicity includes pruritus, rash, or both.

^d^ Pancreatic toxicity includes asymptomatic lipase elevation. irAEs, immune‐related adverse events.

### 3.3 Relationship Between irAEs and Tumor Response

**FIGURE 3:**
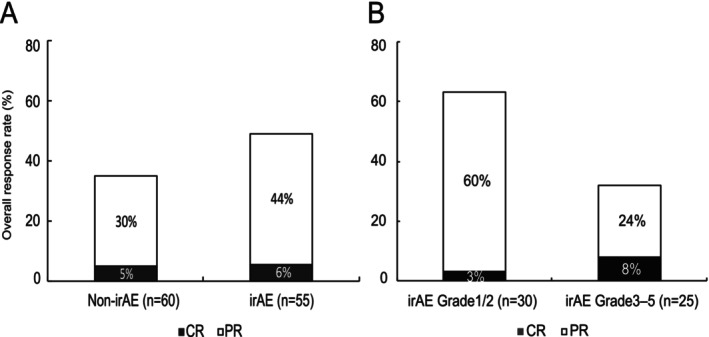
Overall response rate according to (A) the presence or absence and (B) severity of immune‐related adverse events (irAEs). Patients with irAEs were divided into two groups according to the severity of the irAEs, namely Grade 1/2 or Grade ≥ 3, according to the Common Terminology Criteria for Adverse Events (CTCAE) v5.0. CR, complete response; PR, partial response.

The ORR to first‐line therapy was not significantly different between the irAE and non‐irAE groups (50% vs. 35%; *p* = 0.13; Figure 3A). Based on irAE severity, the ORR was 63% in patients with Grade 1/2 irAEs and 32% in those with Grade 3–5 irAEs (Figure 3B). After adjusting for covariates including sex, age, driver mutation status, and ECOG‐PS in a logistic regression analysis, the association between the occurrence of irAEs and ORR remained not significant (*p* = 0.13; Table 3).

**TABLE 3:** Multivariable analysis of the occurrence of any immune‐related adverse event and the effects of covariates on the overall response rate.

	HR (95% CI)	*p*
Occurrence of irAE		
No	1 (reference)	
Yes	0.56 (0.23–1.19)	0.13
Age		
< 75 years	1 (reference)	
≥ 75 years	1.05 (0.45–2.41)	0.92
Sex		
Female	1 (reference)	
Male	0.53 (0.18–1.56)	0.25
Driver mutation		
No	1 (reference)	
Yes	0.75 (0.29–1.91)	0.55
ECOG‐PS		
≥ 2	1 (reference)	
0, 1	0.68 (0.21–2.21)	0.52

Abbreviations: CI, confidence interval; ECOG‐PS, Eastern Cooperative Oncology Group performance status; HR, hazard ratio; irAE, immune‐related adverse event.

### 3.4 Association Between irAEs and Survival Outcomes

**FIGURE 4:**
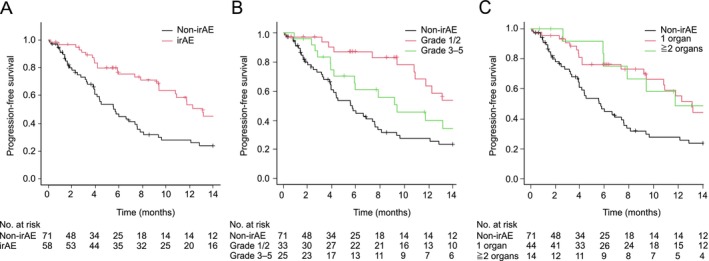
Unadjusted Kaplan–Meier estimates of progression‐free survival (PFS). (A) PFS in patients according to the presence or absence of immune‐related adverse events (irAEs). Median (95% confidence interval [CI]) PFS in the irAE and non‐irAE groups was 5.7 (3.9–7.6) and 13.1 (9.4–17.7) months, respectively. (B) PFS in the non‐irAE group and in patients with Grade 1/2 or Grade 3–5 irAEs; the median (95% CI) PFS in these three groups was 5.7 (3.9–7.6), 14.7 (10.9–20.8), and 9.4 (4.2–16.8) months, respectively. (C) PFS in the non‐irAE group and in patients with irAEs involving one organ or two or more organs; the median (95% CI) PFS in these three groups was 5.7 (3.9–7.6), 13.1 (9.4–17.7), and 11.7 (5.9–N/A) months, respectively. N/A, not applicable.

The median follow‐up duration for all patients was 9.7 months (minimum–maximum 0.3–46.6 months). The median PFS and OS for all patients were 9.2 months (95% CI, 6.0–11.8 months) and 27.3 months (95% CI, 17.9–45.9 months), respectively. The log‐rank test showed significantly longer median PFS in the irAE group (13.1 months; 95% CI, 9.4–17.7 months) than in the non‐irAE group (5.7 months [95% CI, 3.9–7.6 months]; HR 0.55 [95% CI, 0.35–0.86; *p* < 0.01]; Figure 4A). Based on the grade of the most severe irAEs, PFS was significantly longer in patients with Grade 1/2 irAEs than in the non‐irAE group (median PFS 14.7 vs. 5.7 months, respectively; log‐rank *p* = 0.02, post hoc Holm test *p* = 0.02; Figure 4B). The log‐rank test revealed a significant difference in PFS depending on the number of organs affected by irAEs (0, 1, or ≥ 2) in each patient (*p* = 0.03), but post hoc tests did not reveal significant pairwise differences (*p* = 0.07; Figure 4C).

**FIGURE 5:**
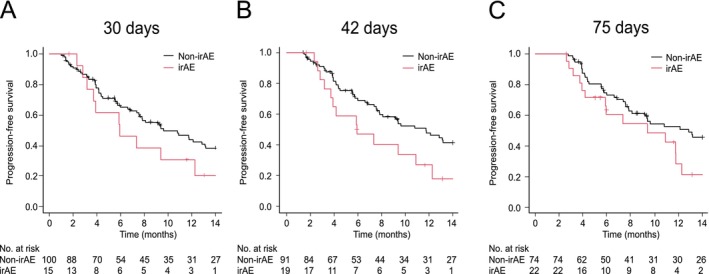
Landmark analyses of progression‐free survival (PFS) in patients with and without immune‐related adverse events (irAEs) analyzed at (A) 30, (B) 42, and (C) 75 days. Median (95% confidence interval) PFS in the non‐irAE and irAE groups was 9.6 (7.6–13.2) and 5.9 (3.2–12.3) months, respectively, at the 30‐day landmark; 11.7 (7.8–16.1) and 5.9 (3.2–10.9) months, respectively, at the 42‐day landmark; and 12.8 (8.1–19.0) and 9.4 (4.2–12.3) months, respectively, at the 75‐day landmark.

To address ITB, landmark analyses were performed at 30, 42, and 75 days. These analyses compared patients who had developed an irAE by the landmark time to those who had not. PFS did not differ significantly between the irAE group and the non‐irAE group at either 30 days (median PFS 5.9 vs. 9.6 months, respectively; *p* = 0.10) or 75 days (9.4 vs. 12.8 months, respectively; *p* = 0.10). However, at the 42‐day landmark, the median PFS was significantly longer in the non‐irAE than irAE group (11.7 vs. 5.9 months, respectively; *p* = 0.02; Figure 5). In the multivariable time‐dependent Cox model, which adjusted for confounders and ITB by treating irAE occurrence as a time‐dependent event, developing an irAE was not significantly associated with PFS (HR 1.16; 95% CI, 0.72–1.88; *p* = 0.53). In contrast, ECOG‐PS 0/1 and PD‐L1 ≥ 50% and combining ICI with chemotherapy was associated with significantly longer PFS than ECOG‐PS ≥ 2 (HR 0.35; 95% CI, 0.17–0.69; *p* = 0.02), PD‐L1 1%–49% (HR 3.20; 95% CI, 1.48–6.90; *p* = 0.003), PD‐L1 < 1% (HR 2.66; 95% CI, 1.42–4.95; *p* = 0.002), and ICI monotherapy (HR 0.34; 95% CI, 0.17–0.66; *p* = 0.002), respectively (Table 4).

**TABLE 4:** Multivariable analysis of the occurrence of any irAE and the effect of covariates on progression‐free survival.

Variable	HR (95% CI)	*p*
Occurrence of irAE		
No	1 (reference)	
Yes	1.15 (0.68–1.95)	0.53
Age		
< 75 years old	1 (reference)	
≥ 75 years old	1.03 (0.56–1.90)	0.75
Sex		
Female	1 (reference)	
Male	0.89 (0.44–1.82)	0.89
Driver mutation		
No	1 (reference)	
Yes	1.11 (0.60–2.05)	0.46
Performance status		
≥ 2	1 (reference)	
0, 1	0.35 (0.17–0.69)	0.02
PD‐L1 status		
≥ 50%	1 (reference)	
1%–49%	3.20 (1.48–6.90)	0.003
< 1%	2.66 (1.42–4.95)	0.002
With or without chemotherapy		
ICI only	1 (reference)	
With chemotherapy	0.34 (0.17–0.66)	0.002

Abbreviations: CI, confidence interval; ECOG‐PS, Eastern Cooperative Oncology Group performance status; HR, hazard ratio; ICI, immune checkpoint inhibitor; irAE, immune‐related adverse event.

**FIGURE 6:**
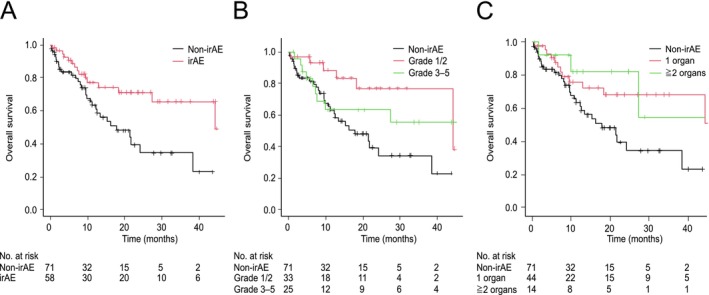
Unadjusted Kaplan–Meier estimates of overall survival (OS). (A) OS in patients according to the presence or absence of immune‐related adverse events (irAEs). Median (95% confidence interval [CI]) OS in the irAE and non‐irAE groups was 17.9 (11.2–38.4) and 44.3 (27.3–N/A) months, respectively. (B) OS in the non‐irAE group and in patients with Grade 1/2 or Grade 3–5 irAEs; the median (95% CI) OS in these three groups was 17.9 (11.2–38.4), 44.3 (44.3–N/A), and 44.3 (7.3–N/A) months, respectively. (C) OS in the non‐irAE group and in patients with irAEs involving one organ or two or more organs; the median (95% CI) OS in these three groups was 17.9 (11.2–38.4), 45.9 (18.3–N/A), and not reached (10–N/A) months, respectively. N/A, not applicable.

Similarly, using the unadjusted analysis, median OS was significantly longer in the irAE group (44.3 months; 95% CI, 27.3 months–not applicable) than non‐irAE group (17.9 months [95% CI, 11.2–38.4 months]; HR 0.42 [95% CI, 0.23–0.81; *p* < 0.01]; Figure 6A). OS was significantly longer among patients with Grade 1/2 irAEs as their most severe event than among the non‐irAE group (median OS 44.3 vs. 17.9 months, respectively; log‐rank *p* = 0.02, post hoc Holm test *p* = 0.01; Figure 6B). OS differed significantly according to the number of affected organs (0, 1, or ≥ 2; log‐rank *p* = 0.03); however, pairwise comparisons did not reveal significant differences after post hoc testing (*p* = 0.06; Figure 6C).

**FIGURE 7:**
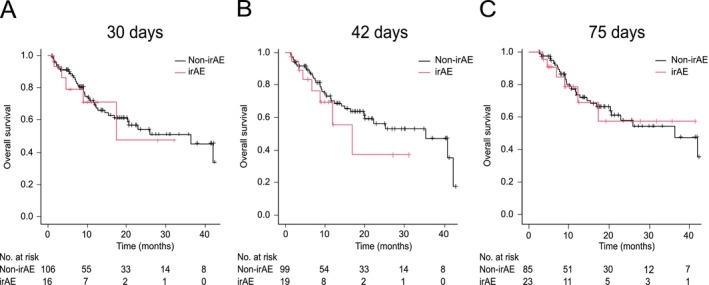
Landmark analyses of overall survival (OS) in patients with and without immune‐related adverse events (irAEs) analyzed at (A) 30, (B) 42, and (C) 75 days. Median (95% confidence interval) OS in the non‐irAE and irAE groups was 38.4 (17.9–45.9) and 18.3 (4.8–N/A) months, respectively, at the 30‐day landmark; 38.4 (21.5–45.9) and 18.3 (7.3–N/A) months, respectively, at the 42‐day landmark; and 38.4 (21.7–45.9) and not reached (12.9–N/A) at the 75‐day landmark. N/A, not applicable.

Using landmark analyses, OS was not significantly different between the irAE and non‐irAE groups at 30 days (median OS 18.3 vs. 38.4 months, respectively; *p* = 0.72), 42 days (median OS 18.3 vs. 38.4 months, respectively; *p* = 0.31), and 75 days (median OS not reached vs. 38.4 months, respectively; *p* = 0.86; Figure 7). In the multivariable time‐dependent Cox model, the occurrence of an irAE was not significantly associated with OS (HR 0.93; 95% CI, 0.48–1.80; *p* = 0.83). However, ECOG‐PS 0/1 remained significantly associated with longer OS (HR 0.19; 95% CI, 0.09–0.40; *p* < 0.01; Table 5).

**TABLE 5:** Multivariable analysis of the occurrence of any immune‐related adverse event and the effects of covariates on overall survival.

	HR (95% CI)	*p*
Occurrence of irAE		
No	1 (reference)	
Yes	0.93 (0.48–1.80)	0.83
Age		
< 75 years	1 (reference)	
≥ 75 years	1.3 (0.67–2.58)	0.45
Sex		
Female	1 (reference)	
Male	0.56 (0.26–1.20)	0.14
Driver mutation		
No	1 (reference)	
Yes	0.69 (0.34–1.44)	0.33
ECOG‐PS		
≥ 2	1 (reference)	
0, 1	0.19 (0.09–0.40)	< 0.01

Abbreviations: CI, confidence interval; ECOG‐PS, Eastern Cooperative Oncology Group performance status; HR, hazard ratio; irAE, immune‐related adverse event.

### 3.5 Association of Corticosteroid Use With Survival

To address the potential impact of immunosuppressive therapy on survival, we evaluated the association between systemic corticosteroid use for irAE management and clinical outcomes. Among the 58 patients who developed irAEs, 31 (53.4%) received systemic corticosteroids. Fisher's exact test showed that patients with Grade 3–5 irAEs were significantly more likely to require corticosteroids compared to those with Grade 1/2 irAEs (64.5% vs. 18.5%, *p* < 0.001). Notably, the median OS was significantly shorter in patients treated with corticosteroids compared to those who were not (median OS 27.0 months vs. not reached, respectively; *p* < 0.001; Figure S1). Since there was no significant difference in OS between patients with Grade 1/2 and those with Grade 3–5 irAEs (*p* = 0.38, Figure 6B), the shorter survival observed in the corticosteroid group suggests that the use of corticosteroids itself, or the subsequent discontinuation of ICI treatment, may have adversely affected survival outcomes. In an exploratory multivariable time‐dependent Cox model including corticosteroid use as a covariate, the development of irAEs remained statistically insignificant for OS (HR 0.80; 95% CI, 0.38–1.70; *p* = 0.56, data not shown), suggesting that the lack of survival benefit associated with irAEs was consistent even after accounting for the negative impact of corticosteroids.

## 4 Discussion

In this retrospective cohort study, we investigated the association between irAEs and ORR, PFS, and OS in patients receiving first‐line ICIs for advanced NSCLC at Saitama Medical Center, Saitama Medical University. To accurately evaluate the association between time‐varying events like irAEs and survival outcomes, ITB should be considered and addressed. Therefore, we used landmark analysis and a time‐dependent Cox model in the present study. Unlike many previous studies including various treatment lines, as shown in Table 6, our focus on first‐line therapy provides data more reflective of current routine clinical practice. Consequently, the importance of using these appropriate methods was highlighted. In this study, the results of the landmark analyses were largely consistent with those of the time‐dependent Cox model, with the only significant difference being in PFS at the 42‐day landmark, whereby PFS was longer in the non‐irAE than in the irAE group. When treating the development of irAEs as a time‐dependent event to account for ITB and adjusting for confounders, neither method showed a significant association between the occurrence of irAEs and improved PFS or OS. This consistency strengthens the conclusion that, in this cohort, irAE occurrence was not independently associated with better prognosis.

**TABLE 6:** Summary of previous studies investigating immune‐related adverse events and survival in nonsmall cell lung cancer with immortal time bias adjustment.

Study (year)	Treatment line	ICI regimen	No. of patients	ITB adjustment method(s)	Association of irAEs with PFS or OS
Fujimoto et al. (2018) [19]	Second line or later	Monotherapy	613	Landmark (2‐mo)	Negative (PFS)
Haratani et al. (2018) [13]	Second line or later	Monotherapy	134	Landmark (6‐wk)	Positive (PFS, OS)
Sato et al. (2018) [20]	Second line or later	Monotherapy	38	Landmark (60‐d)	Negative (PFS)
Noguchi et al. (2020) [21]	Any line	Monotherapy	94	Landmark (9‐wk)	Negative (PFS, OS)
Maillet et al. (2020) [22]	Any line	Monotherapy	410 (including 304 NSCLC)	Time‐dependent Cox analysis	Positive (PFS, OS)
Riudavets et al. (2020) [14]	Any line	Alone or with chemotherapy	267	Landmark (PFS: 2.4, 6, 12‐mo; OS: 5.9, 6, 12‐mo)	Positive (PFS, OS)
Kfoury et al. (2022) [18]	Any line	Monotherapy	577 (including 299 NSCLC)	Landmark (12‐wk) and time‐dependent Cox analysis	TD Cox: Positive (PFS, OS) Landmark: Negative
Kurokawa et al. (2022) [15]	First line	Alone or with chemotherapy	148	Landmark (12‐wk)	Alone: Positive (PFS) With chemotherapy: Negative (PFS)
Cook et al. (2024) [16]	Any line	Alone or with chemotherapy	803	Landmark (12‐wk)	Positive (OS)
Yu et al. (2024) [17]	Any line	Alone or with chemotherapy	425	Landmark (2, 3, 6, 9‐mo) and time‐dependent Cox analysis	Positive (PFS, OS)
Present study	First line	Alone or with chemotherapy	129	Landmark (30, 42, 75‐d) and time‐dependent Cox analysis	Negative (PFS, OS)

Abbreviations: d: days, irAEs: immune‐related adverse events, ITB: immortal time bias, mo: months, NSCLC: nonsmall cell lung cancer, TD Cox: time‐dependent Cox analysis, wk: weeks.

Poor performance status is known as a negative prognostic factor. Our multivariable analyses confirmed that ECOG‐PS ≥ 2 was independently associated with significantly shorter PFS and OS. This finding aligns with those of previous studies [16, 18, 19, 21, 24, 25]. Our results suggest that the apparent survival benefit linked to irAEs reported in the initial unadjusted analyses may be confounded by factors like better baseline performance status, in addition to the significant impact of ITB. Performance status remains a critical prognostic factor in patients receiving ICIs.

PD‐L1 expression ≥ 50% was independently associated with significantly longer PFS compared with PD‐L1 expression of 1%–49% or < 1%. High PD‐L1 expression is a well‐established predictor of improved outcomes with ICI therapy, and our findings are consistent with previous reports demonstrating superior survival in this subgroup [26, 27].

ICI monotherapy was independently associated with significantly shorter PFS compared with ICI with chemotherapy. This result likely reflects the therapeutic contribution of cytotoxic chemotherapy in prolonging PFS. Indeed, in a multivariable Cox proportional hazards model in which driver mutation status was replaced with treatment modality (ICI with chemotherapy vs. ICI monotherapy), the ICI with chemotherapy group did not show a statistically significant OS benefit over ICI monotherapy (HR, 0.51; 95% CI, 0.24–1.06; *p* = 0.07).

The impact of irAE management, particularly corticosteroid use, on survival outcomes remains a subject of debate. In our cohort, systemic corticosteroid use was significantly associated with shorter OS. Although high‐grade irAEs were more frequent in the corticosteroid‐treated group, survival did not differ according to irAE grade, suggesting that the poor prognosis observed in corticosteroid‐treated patients may be attributable to the immunosuppressive effects of corticosteroids themselves or to subsequent discontinuation of ICI therapy. This finding aligns with previous studies suggesting that high‐dose corticosteroids or permanent discontinuation of ICIs due to toxicity may compromise antitumor efficacy [14]. Therefore, careful management of irAEs is crucial to balancing toxicity control with the preservation of the anti‐tumor immune response.

This study has several limitations. First, it was a single‐center, retrospective analysis. The patient population in our single institution might possess specific characteristics that limit the external validity of our findings. Furthermore, the limited sample size (*N* = 129) and, more importantly, the small number of events for OS (*n* = 46) resulted in insufficient statistical power. Given the lack of a robust validation method for time‐dependent Cox models, a post hoc power calculation, based on Schoenfeld's method, revealed that our study had only 22.5% power to detect a moderate clinically relevant effect (hazard ratio [HR] = 0.70) for OS at an *α* level of 0.05 while the power to detect a stronger effect [HR = 0.60] was 40.7%. The PFS analysis (*n* = 79 events) was similarly underpowered with only 35.1% power to detect the same effect (HR = 0.70) while the power to detect a stronger effect (HR = 0.60) was 61.7%. This suggests that our negative finding regarding the association between irAEs and OS (HR 0.93) is likely to be the result of a Type II error, and a moderate survival benefit cannot be ruled out. Second, despite the use of two methods (landmark and time‐dependent Cox) to mitigate the immortal time bias, new selection or detection biases may have been introduced, and due to the retrospective nature, unmeasured confounders could not be completely accounted for. Third, we did not evaluate the potential impact of key confounding factors such as the detailed dose and duration of corticosteroid use for irAE management, or the association between specific types of irAEs and outcomes. Given these limitations, our findings should be interpreted with caution. Large‐scale, multicenter prospective studies are warranted to confirm the relationship between irAEs and ICI efficacy.

In conclusion, after appropriate adjustment for ITB using landmark and time‐dependent Cox analyses, our study found no significant association between the development of irAEs and ORR, PFS, or OS in patients with advanced NSCLC receiving first‐line ICIs. Good baseline ECOG‐PS was independently associated with improved survival outcomes.

## Author Contributions

**Yoshiki Kuwabara:** conceptualization. **Yoshiki Kuwabara:** formal analysis. **Yoshiki Kuwabara** and **Hiroyuki Ohya:** investigation. **Yoshiki Kuwabara**, **Maiko Osawa**, **Yohei Kawasaki:** methodology. **Yoshiki Kuwabara**, **Kota Shiraishi**, **Itsuka Matsumoto**, **Shigeru Ishii**, **Shin Yokosuka**, **Masatoshi Abe**, **Tomoyuki Takahashi**, **Yuichiro Kawano**, **Hiroaki Nishimura**, **Maiko Toda‐Sasaki**, **Yumiko Kobayashi‐Ogawa**, **Satoshi Kikuchi**, **Yusuke Hirata**, **Kosuke Sakai**, **Hiroyuki Kyoyama**, **Gaku Moriyama**, **Nobuyuki Koyama**, **Kazutsugu Uematsu:** resources. **Kazutsugu Uematsu**, **Maiko Osawa**, **Yohei Kawasaki:** supervision. **Yoshiki Kuwabara:** visualization. **Yoshiki Kuwabara:** writing – original draft. **Kazutsugu Uematsu:** writing – review and editing.

## Funding

The authors have nothing to report.

## Ethics Statement

The study protocol was approved by the Ethics Board of Saitama Medical Center, Saitama Medical University (Approval no. 2024‐219).

## Consent

This study is a retrospective study, and patient consent was obtained through the facility's opt‐out procedure.

## Conflicts of Interest

Y. Kuwabara has received honoraria from AstraZeneca K.K. and Chugai Pharmaceutical Co. T.T. has received honoraria from AstraZeneca K.K. Y.K.‐O. has received honoraria from Daiichi Sankyo Co. Ltd. Y.H. has received honoraria from AstraZeneca K.K. K. Sakai has received honoraria from AstraZeneca K.K., Chugai Pharmaceutical Co., Nippon Boehringer Ingelheim Co., Novartis Pharma K.K., Pfizer Japan Inc., Eli Lilly Japan K.K., Bristol‐Myers Squibb K.K., Nippon Kayaku Co. Ltd., Merck Biopharma Co. Ltd., and Thermo Fisher Scientific Inc., as well as research funding from Eli Lilly Japan K.K. H.K. has received honoraria from AstraZeneca K.K., Chugai Pharmaceutical Co., Bristol‐Myers Squibb K.K., Nippon Boehringer Ingelheim Co., and Takeda Pharmaceutical Co. Ltd. G.M. has received honoraria from AstraZeneca K.K., Chugai Pharmaceutical Co., Eli Lilly Japan K.K., Merck Biopharma Co. Ltd., and MSD K.K. N.K. has received honoraria from AstraZeneca K.K., Chugai Pharmaceutical Co., Nippon Boehringer Ingelheim Co., Novartis Pharma K.K., Eli Lilly Japan K.K., Bristol‐Myers Squibb K.K., Amgen K.K., Nippon Kayaku Co. Ltd., TAIHO Pharmaceutical Co., Daiichi Sankyo Co. Ltd., Takeda Pharmaceutical Co. Ltd., and MSD K.K. K.U. has received honoraria from AstraZeneca K.K., Chugai Pharmaceutical Co., Nippon Boehringer Ingelheim Co., Eli Lilly Japan K.K., Novartis Pharma K.K., TAIHO Pharmaceutical Co., Bristol‐Myers Squibb K.K., and MSD K.K., as well as research funding from Chugai Pharmaceutical Co., Nippon Boehringer Ingelheim Co., Novartis Pharma K.K., and TAIHO Pharmaceutical Co. The other authors declare no conflicts of interest.

## Supporting information


**Figure S1:** Unadjusted Kaplan–Meier estimates of overall survival (OS) in patients with immune‐related adverse events.

## Data Availability

The data that support the findings of this study are available from the corresponding author upon reasonable request.
