# Supplementary figures and images for: Association of Immune‐Related Adverse Events and the Efficacy of Immune Checkpoint Inhibitors in Non‐Small Cell Lung Cancer: Adjusting for Immortal Time Bias
Source: Thorac Cancer. 2026 Mar 16;17(6):e70261. doi: 10.1111/1759-7714.70261 (PMC13097447; doi:10.1111/1759-7714.70261)

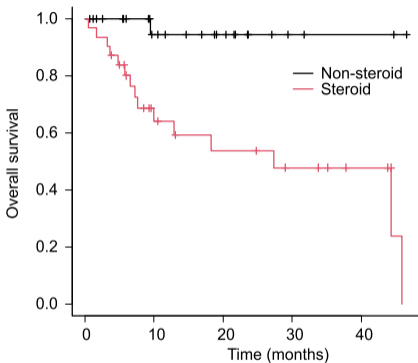

No. at risk  
Non-steroid  
Steroid

27

16

10

3

2

31

14

10

7

4

Supplement: Supplementary file 1 — Figure S1: Unadjusted Kaplan–Meier estimates of overall survival (OS) in patients with immune‐related adverse events. [file TCA-17-e70261-s001.pdf]
